# Cognitive and mood improvements following acute supplementation with purple grape juice in healthy young adults

**DOI:** 10.1007/s00394-017-1454-7

**Published:** 2017-04-20

**Authors:** C. F. Haskell-Ramsay, R. C. Stuart, E. J. Okello, A. W. Watson

**Affiliations:** 1 Brain, Performance and Nutrition Research Centre, Northumbria University, Newcastle upon Tyne, NE1 8ST UK; 2 School of Agriculture, Food and Rural Development, Newcastle University, Newcastle upon Tyne, NE1 7RU UK

**Keywords:** Cognition, Cognitive, Mood, Grape, Phenolic, Polyphenol, Phytochemical

## Abstract

**Purpose:**

Berry-derived phenolic compounds found in grapes have been associated with a number of health benefits, including the augmentation of human brain function and cognition. Previous intervention studies of Concord grape juice have demonstrated improvement to memory and driving ability following 3- to 4-month supplementation in middle-aged and older adults. However, no studies to date have demonstrated acute cognitive benefits of grape juice, and investigation of these effects in young adults is lacking.

**Methods:**

This randomised, placebo-controlled, double-blind, counterbalanced-crossover study, assessed the effects of 230 ml purple grape juice or sugar-matched control in 20 healthy young adults. Computerised measures of episodic memory, working memory, attention and mood were completed at baseline and following a 20-min absorption period.

**Results:**

Purple grape juice significantly improved reaction time on a composite attention measure (*p* = 0.047) and increased calm ratings (*p* = 0.046) when compared to placebo. Order effects also indicated an enduring positive effect on pre-dose memory reaction time (*p* = 0.018) and post-dose calm ratings (*p* = 0.019) when purple grape was consumed first.

**Conclusions:**

These findings in a small sample of healthy young adults suggest that purple grape juice can acutely enhance aspects of cognition and mood. No significant effects of juice were observed on memory measures, suggesting that these may be less susceptible to manipulation following acute supplementation in healthy young adults. Potential mechanisms underlying these effects include modulation of cerebral blood flow, glucoregulation and inhibition of monoamine oxidase activity, all of which require further exploration.

## Introduction

Phenolic compounds are found in varying concentrations in a range of plant-based food sources, such as legumes, fruit, vegetables, herbal extracts, spices, coffee, tea and cocoa. These compounds are grouped into phenolic acids, stilbenes, lignans and flavonoids on the basis of the number of phenol rings; with flavonoids further sub-classified into flavonols, flavones, isoflavones, flavanones, anthocyanidins and flavan-3-ols as a function of their structural complexity [1]. Berry fruits, such as strawberry, blueberry, blackcurrant and grape, represent a rich source of (poly)phenols, which are linked to a number of health benefits including modulation of inflammation [2, 3], reductions in risk of cardiovascular disease (see [4] for systematic review), anti-cancer effects [5], and protection against neurodegenerative diseases [6–8]. In particular, commercially available purple grape juice, containing 65% Concord grape, was shown to have the highest concentration of (poly)phenols relative to other commercially available fruit juices, as well as a competitively high diversity of individual phenolic compounds [9, 10]. Concord grapes (*Vitis labrusca*) contain a unique mixture of flavan-3-ols, tartaric esters of hydroxycinnamates, and anthocyanins; the latter of which comprise 46% of the total phenolic content [9, 10].

Recent systematic reviews of epidemiological and intervention studies suggest a beneficial role for berry (poly)phenols in relation to cognition [11, 12]. These are supported by evidence of a number of potential direct and indirect mechanisms, including their interaction with gut microbiota [13], modulation of neuroinflammation [14], improved cerebrovascular function (reviewed in [15]), modulation of glucoregulation [16] and increased spine density and neurogenesis, particularly in the hippocampus [17]. Flavonoids, at low nanomolar concentrations, also induce synaptic plasticity (for review see [18]) due to modulation of receptor function and gene expression and via interaction with signalling pathways through a number of potential actions including binding to ATP sites, regulation of kinase activity and modulation of transcription factor activation and binding [19, 20]. Specific studies of Concord grape juice (CGJ) are also beginning to emerge, suggesting a positive impact on cognition following short-term supplementation. In the first of two parallel groups studies in older adults with mild cognitive impairment (*N* = 12), improvements to California Verbal Learning Test (CVLT) list acquisition were observed following 12-week supplementation with 6–9 ml/kg per day of CGJ (range 444–621 ml/day), when compared to placebo [21]. A similar dosing schedule (range 355–621 ml/day) led to reduced CVLT recognition memory errors and increased activation in the right superior parietal cortex and right middle frontal cortex following 16-week supplementation (*N* = 21) [22]. A recent crossover study demonstrated improvements to visuo-spatial learning and driving performance following a 12-week intervention with CGJ in 40- to 50-year-old healthy working mothers of preteens. There were also a number of interaction effects with study phase indicating carryover effects of grape juice. Specifically, consumption of CGJ in arm 1 was associated with enduring benefits to verbal recall, executive function and driving ability in the second arm of the study when placebo was consumed [23].

The only study to date to assess cognitive effects following a single serve of purple grape juice [24], failed to demonstrate effects of 10 ml/kg of CGJ in relation to implicit memory, mood and appetite in 18- to 50-year-old smokers (mean age 26 ± 7.5 years). The lack of effects in this study may relate to task selection, as there is no evidence for modulation of implicit memory by phenolic compounds from the available literature. It is also pertinent to note that previous studies in young adults have demonstrated acute cognitive benefits of (poly)phenols following supplementation of cocoa flavan-3-ols [25] and blackcurrant [26]. Effects of (poly)phenols on subjective state have also been observed in healthy young adults following acute supplementation with cocoa [25] and orange juice [27], and it is important that these effects are explored further given the well-established relationship between cognition and mood (e.g. see [28] for review). In light of this, the current randomised, placebo-controlled, double-blind, counterbalanced-crossover study examined the acute cognitive and mood effects of a 230 ml single serving of commercially available purple grape juice in healthy young adults.

## Method

### Design

A randomised, placebo-controlled, double-blind, counterbalanced-crossover design was employed. The study was approved by Northumbria University’s Department of Psychology Ethics Committee and was conducted in accordance with the Declaration of Helsinki.

### Participants

Twenty participants (7 males; mean age 21.05 years, SD 0.89) were drawn through an opportunity sample within Newcastle upon Tyne and the surrounding areas. Due to previous studies showing acute benefits of (poly)phenols in healthy young adults [24, 25], and a lack of research into effects of purple grape juice in this population, the current study assessed effects in a healthy young adult sample. All participants were aged 18–35 years. Female participants were not pregnant or seeking to become pregnant, or lactating. All participants had no pre-existing medical conditions or history of neurological, vascular or psychiatric illness; had no current or historical diagnosis of drug or alcohol abuse; were not currently taking medication (excluding the contraceptive pill) or dietary supplements; did not smoke >3 cigarettes per day or consume high levels of caffeine (>400 mg/day); did not suffer food allergies or sensitivities; and English was their first language. No payment was made for participation.

### Treatment

Placebo and active treatment were administered as 230 ml drinks. The active treatment consisted of 200 ml Welch’s™ purple grape juice (based upon single serving guidelines at the time of the study) plus 30 ml of Schweppes™ blackcurrant flavour cordial (containing 14 kcal and 3.1 g sugar per 100 ml). Placebo consisted of 200 ml Welch’s™ white grape juice plus 10 ml blackcurrant flavour cordial and 20 ml cold water. Schweppes™ blackcurrant flavour cordial was added at different volumes to the active and placebo drinks to ensure matched sugar content, this also served to mask the flavour of the drinks. On the day of testing, beverages were prepared by an independent third party and administered in an opaque container to prevent any visual differences being detected. In order to confirm the phenolic content of the two drinks, analyses of anthocyanin and total phenolic content were conducted as described below. Composition data can be found in Table 1.

**Table 1:** Nutritional content of drinks

	PGJ	WGJ	Active	Placebo
Energy (kcal)	136	138	140.2	139.4
Sugars (g)	33	33.6	33.9	33.9
Anthocyanin content (mg/l)^#^	136.6	0.34	138.3	1.04
Phenolic content (μg/ml)*	1681.7	100	1504.5	135.1

*PGJ* pure purple grape juice (200 ml), *WGJ* pure white grape juice (200 ml), *Active* active drink administered in the study—PGJ + 30 ml blackcurrant cordial, *Placebo* WGJ + 10 ml blackcurrant cordial + 20 ml water

^#^Total monomeric anthocyanin content expressed as cyanidin-3-glucoside equivalents

* Total phenolic content expressed as gallic acid equivalents

### Determination of anthocyanin content

Total monomeric anthocyanin content was determined by the pH differential method (AOAC Official Method 2005.2) [29]. Briefly, two dilutions of the fruit juices were prepared, one in a potassium chloride buffer (pH 1.0) and the other in a sodium acetate buffer (pH 4.5) and absorbance measured at 520 and 700 nm within 20-min of incubation at room temperature. The anthocyanin pigment concentration was calculated and expressed as mg/L cyanidin-3-glucoside equivalents using the following equation:

$${\text{anthocyanin pigment }} = \frac{{A \times {\text{MW}} \times {\text{DF}} \times 10^{3} }}{\varepsilon \times 1}$$

where *A* = (A520 − A700 nm) pH 1.0 − (A520 − A700 nm) pH 4.5; MW is the molecular weight for cyanidin-3-glucoside (449.2); DF is the dilution factor; 1 is the path length in cm of the cuvette/spectrophotometer; *ε* = 26,900 is the extinction coefficient, in L × mol^−1^ × cm for cyanidin-3-glucoside and 10^3^ is the factor for converting from g to mg.

### Determination of total phenolic content

The phenolic content in the samples was determined using the Folin–Ciocalteu methods in a 96-well microplate according to the procedure by Zhang et al. [30]. A gallic acid standard curve (3.125–100 µg/ml) against a blank at 765 nm was used to calculate the phenolic content as gallic acid equivalents (GAE µg/ml). Calculation of content took into account the dilution factor (DF) for each juice.

### Cognitive and mood measures

All cognitive and mood measures were administered using the Computerised Mental Performance Assessment System (COMPASS, Northumbria University, Newcastle upon Tyne, UK), a software platform for the presentation of classic and bespoke computerised cognitive tasks. This platform has previously been shown to be sensitive to a range of nutritional interventions [31–33], including blackcurrant supplementation [26]. The computerised tests were conducted via a laptop computer with all stimuli randomised across participant and assessment. Progress through the tasks was controlled by the participant, with brief instructions given on-screen before the start of each task. Tasks were presented in the same order on each occasion and, with the exception of the paper and pencil tasks (word recall and delayed word recall), responses were made using a response pad. Due to the limited available data regarding the effects of acute consumption of purple grape juice on cognitive performance outcomes, the tasks selected comprised standard psychometric tasks assessing cognitive function across different domains. The entire selection of tasks took approximately 20-min to complete. See Table 2 for order, description and scoring of tasks, as well as domain ascribed (see [34]).

**Table 2:** Cognitive tasks in order of presentation

Task	Descriptor	Scoring	Domain
Word presentation	A list of words is displayed on the screen, one word at a time. In this case, 15 words were presented with a display time of 1 s and inter-stimulus interval of 1 s	–	
Immediate word recall	Participants are instructed to write down the words that were presented. In this case, 60 s were given to complete the task	% of words correctly recalled	Episodic memory
Picture presentation	A series of photographic images are displayed on the screen, one at a time. In this case, 20 images were presented with a display time of 2 s and an inter-stimulus interval of 1 s	–	
Simple reaction time	An upwards pointing arrow is displayed on the screen at irregular intervals. Participants must respond as quickly as they can as soon as they see the arrow appear. In this case, 50 stimuli were presented	Reaction time (ms)	Attention
Digit vigilance	A fixed number appears on the right of the screen and a series of changing numbers appear on the left of the screen at the rate of 150 per minute. Participants are required to make a response when the number on the left matches the number on the right. In this case the task lasted for 3-min	Accuracy (%) and reaction time for the correct responses (ms)	Attention
Choice reaction time	Arrows pointing left and right appear on the screen at irregular intervals. The participant is required to indicate the direction of the arrow as quickly as possible whenever an arrow is displayed, by pressing the corresponding button. In this case, 50 stimuli were presented	Accuracy (%) and reaction time for the correct responses (ms)	Attention
Numeric working memory	A series of numbers are displayed on the screen, one at a time. Participants are required to memorise these numbers as they appear. Once the series is complete, numbers are displayed one at a time and participants are required to indicate if each number was presented in the previous list or not. In this case, three trials were completed with five target numbers in each trial	Accuracy (%) and reaction time for the correct responses (ms)	Working memory
Delayed word recall	Participants are instructed to write down the words that were presented to them at the beginning of the assessment. In this case, 60 s were given to complete the task	% of words correctly recalled	Episodic memory
Delayed word recognition	All target words that were shown during Word presentation plus an equal number of decoys are displayed on the screen one at a time. Participants indicate if they remember seeing the word earlier or not	Accuracy (%) and reaction time for the correct responses (ms)	Episodic memory
Delayed picture recognition	All target pictures shown during Picture presentation plus an equal number of decoys are displayed on the screen one at a time. Participants indicate if they remember seeing the picture earlier or not	Accuracy (%) and reaction time for the correct responses (ms)	Episodic memory

#### Composite scores

In order to reduce the number of comparisons, *z*-scores were calculated for all cognitive outcomes by pooling data from all assessments as previously described [33]. The tasks were then divided based on whether they represented a memory task (immediate and delayed word recall, numeric working memory, word recognition, picture recognition) or an attention task (simple reaction time, choice reaction time, digit vigilance). These were then further sub-divided on the basis of whether they measured accuracy (% accuracy) or reaction time (ms). This created four composite scores:

Memory accuracy = (^z^immediate word recall + ^z^delayed word recall + ^z^delayed word recognition + ^z^delayed picture recognition + ^z^numeric working memory)/5.

Memory reaction time (RT) = (^z^RT delayed word recognition + ^z^RT delayed picture recognition + ^z^RT numeric working memory)/3.

Attention accuracy = (^z^choice reaction accuracy + ^z^digit vigilance accuracy)/2.

Attention RT = (^z^simple reaction time + ^z^choice reaction time + ^z^digit vigilance reaction time)/3.

#### Subjective mood

*Bond–Lader mood scales* [35] A series of visual analogue scales were completed at the end of the cognitive assessment. Sixteen bipolar lines anchored at each end by an adjective describing a mood (e.g. tense/relaxed) were presented and participants selected a point on the scale that represented how they were feeling at that point in time. Individual item scores were calculated as % distance along the line from the left. The individual scales were combined as recommended by the authors to form three mood factors: alert, calm, content.

### Procedure

Participants were required to attend two 90-min sessions at the Brain, Performance and Nutrition Research Centre at Northumbria University, which took place between 1 and 3 pm in the afternoon. Sessions were conducted between 6 and 7 days apart to ensure sufficient wash out between conditions. All testing took place in a suite of dedicated temperature-controlled university laboratories with participants visually isolated from each other and wearing noise-reduction headphones to decrease the impact of any auditory distractions. All participants were asked to consume the same breakfast and lunch on both study days, and were instructed to fast for at least 1 h, and to abstain from caffeine for at least 2 h, prior to testing. A food diary was utilised to establish compliance to these instructions. Occasional smokers were asked to abstain from smoking on the day of testing. Prior to arrival, participants were randomly allocated to one of two treatment orders as selected through a Latin square.

Following arrival at the laboratory, volunteers were initially briefed and provided written informed consent and their eligibility to participate was confirmed. Participants were then required to provide age and sex information and to complete a food diary. In order to establish baseline performance for that day, participants initially completed a pre-dose 20-min assessment of cognition and mood. This was followed immediately by ingestion of their juice for that day; 5-min was allowed for consumption of the juice through a straw. Following a 20-min absorption period, participants completed the post-dose assessment of cognition and mood. The second study visit was completed at the same time of day for each participant and was identical with the exception of the juice consumed.

### Statistics

In order to minimise the number of comparisons, cognitive scores were collapsed into four composite scores as described above (accuracy for attention tasks, reaction time for attention tasks, accuracy for memory tasks, reaction time for memory tasks) and mood scales were combined to form three factors as recommended by the authors (calm, content, alert) [35].

To assess the possibility of any pre-treatment differences in cognitive performance or mood, baseline data were analysed with linear mixed models including the terms treatment, order, and treatment × order as fixed effects. Post-dose data were then analysed with linear mixed models including the respective baseline as a covariate and the terms treatment, order, and treatment × order as fixed effects. Significant interactions were further explored with pairwise comparisons. Finally, in order to ascertain if any effects on cognition were related to effects on mood, and vice versa, a bivariate Pearson correlation was conducted on change in composite cognitive scores and change in mood ratings for each treatment condition. All data were analysed using SPSS version 22 for windows.

## Results

Mean pre-dose baseline and post-dose scores for each treatment are presented in Table 3, along with standard deviations, *F*, and *p* values. Data capture errors with two participants’ data during the placebo arm and two participants’ data during the purple grape arm resulted in only 16 datasets being analysed for attention measures.

**Table 3:** Unadjusted baseline and post-dose mean and SD for composite *z*-scores and mood ratings

Measure	Treatment	*N*	Baseline				Post-dose				
			Mean	SD	*F*	*p*	Mean	SD	*F*	*p*	
Memory accuracy (%)	Placebo	20	0.27	0.5	1.74	0.204	−0.26	0.59	2.03	0.173	Treatment
	Purple grape	20	0.11	0.48	1.38	0.256	−0.12	0.65	0.08	0.787	Order
					0.07	0.787			1.69	0.212	Treatment × order
Memory RT (ms)	Placebo	20	0.03	0.82	1.64	0.217	−0.17	0.82	0.09	0.770	Treatment
	Purple grape	20	0.22	1.02	0.65	0.431	−0.09	0.70	0.04	0.841	Order
					5.73	0.028			0.54	0.474	Treatment × order
Attention accuracy (%)	Placebo	16	−0.04	0.85	0.11	0.744	0.08	0.69	0.56	0.472	Treatment
	Purple grape	16	−0.08	0.71	4.82	0.046	0.14	0.45	0.43	0.525	Order
					0.54	0.473			0.10	0.763	Treatment × order
Attention RT (ms)	Placebo	16	−0.19	0.63	0.27	0.609	0.13	0.77	4.79	0.047	Treatment
	Purple grape	16	−0.13	0.66	0.30	0.593	−0.18	0.87	0.87	0.367	Order
					1.09	0.313			0.06	0.811	Treatment × order
Bond–Lader Alert	Placebo	20	46.1	16	2.09	0.165	50.5	14.7	0.45	0.513	Treatment
	Purple grape	20	52.6	11.1	0.53	0.474	50.9	14.2	0.02	0.893	Order
					0.57	0.462			0.04	0.836	Treatment × order
Bond–Lader Content	Placebo	20	61.7	14	0.80	0.382	60.3	13.3	0.12	0.737	Treatment
	Purple grape	20	65	13.6	0.04	0.842	63.8	13.6	2.62	0.124	Order
					0.53	0.476			1.59	0.223	Treatment × order
Bond–Lader Calm	Placebo	20	64.2	18.2	1.23	0.281	58.7	12.6	4.57	0.046	Treatment
	Purple grape	20	59.1	16.2	0.25	0.625	62.1	12.2	6.69	0.019	Order
					0.67	0.425			1.62	0.220	Treatment × order

### Baseline scores

There were no statistically significant differences between treatments at baseline. However, there was a main effect of order on attention accuracy [*F*(1, 14) = 4.82, *p* = 0.046]. This indicated that those who consumed placebo first performed better at baseline on both visits (*M* = 0.30, SE = 2.35), compared to those who received purple grape first (*M* = −0.43, SE = 2.35). There was also a treatment × order interaction effect on memory reaction time [*F*(18) = 5.73, *p* = 0.028]. Pairwise comparisons revealed that whilst there was no significant difference between visits in those who consumed placebo first, those who received purple grape first performed better at baseline on visit 2 (*M* = −0.30, SE = 0.29) than visit 1 (*M* = 0.25, SE = 0.29) (*p* = 0.018).

### Post-dose scores

#### Attention RT

**Fig. 1:**
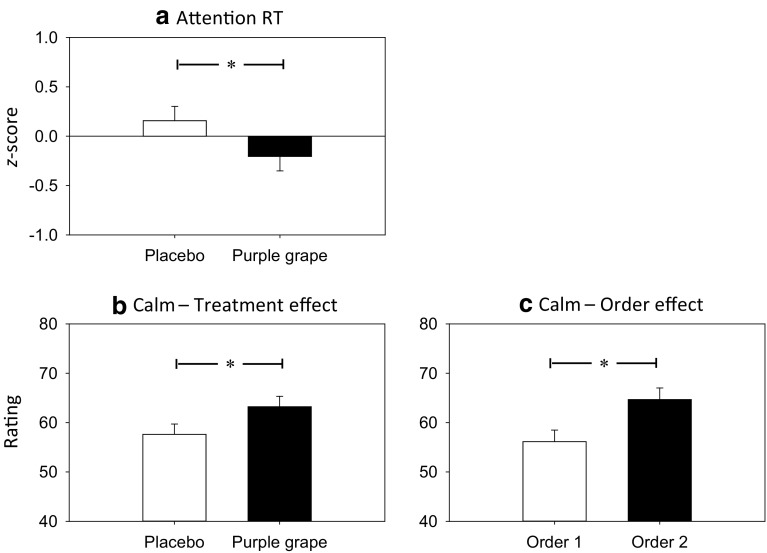
Adjusted means and SE for treatment effects on **a** attention RT and **b** calm ratings and for order effects on **c** calm ratings following placebo and purple grape juice (*RT* reaction time; *p < 0.05; order 1 = placebo then purple grape, order 2 = purple grape then placebo)

A significant main effect of treatment [*F*(1, 13.54) = 4.79, *p* = 0.047] was evinced on the reaction time for attention tasks composite *z*-score. Examination of means revealed that participants were significantly faster following consumption of purple grape (*M* = −0.21, SE = 0.15) relative to placebo (*M* = 0.16, SE = 0.15) (see Fig. 1a).

#### Calm ratings

A significant main effect of treatment on calm ratings [*F*(1, 18.37) = 4.57, *p* = 0.046] was found to be due to significantly higher calm ratings following consumption of purple grape (*M* = 63.22, SE = 2.10) compared to placebo (*M* = 57.61, SE = 2.10); see Fig. 1b. A significant main effect of order was also evinced [*F*(1, 17.82) = 6.69, *p* = 0.019], which indicated that post-dose calm ratings were significantly higher in those who received purple grape first (*M* = 64.68, SE = 2.33), than those who consumed placebo first (*M* = 56.15, SE = 2.33) (see Fig. 1c).

There were no other significant effects of treatment.

### Correlation between cognitive and mood scores

No significant correlations between change in mood ratings and composite cognitive scores were observed.

## Discussion

The results of the current study demonstrate for the first time, that acute consumption of purple grape juice can improve aspects of cognitive performance and mood in healthy young adults. Compared to the sugar-matched, low (poly)phenol control drink, purple grape juice enhanced overall speed on attention tasks and significantly increased calm ratings.

This is the first study to explore acute effects of purple grape on attention and adds to a growing body of evidence demonstrating improved attention task performance following acute supplementation with (poly)phenol-rich foods. Specifically, blackcurrant has been observed to decrease digit vigilance reaction time following a cold-pressed juice extract and to improve accuracy of rapid visual information processing (RVIP) following a freeze-dried extract in young healthy adults [26]. Similarly, cocoa consumption was shown to improve serial three subtraction performance in healthy young adults, with a dose-specific increase to speed of RVIP [25]. However, a recent 12-week intervention in 40- to 50-year-old mothers failed to find an effect of Concord grape juice (CGJ) on RVIP [23] raising the possibility that this effect is only seen in younger adults or is modified following repeated consumption. Recent data demonstrating improved continuous performance task accuracy and increased simple finger tapping following acute orange juice in middle-age males [27] would argue for the latter of these suggestions and this is supported by evidence for acute improvement to digit vigilance accuracy and serial three subtraction performance in elderly adults following curcumin consumption [36]. However, differences in methodology make direct comparisons difficult and further work is required exploring effects on attention in older adults, as this is often overlooked in favour of memory given its susceptibility to ageing. The lack of effects on memory in the current study is interesting as acute supplementation with wild blueberry has been shown to improve memory in 7- to 10-year-old children [37], whereas the studies described above in middle-aged [27] and elderly participants [36] failed to find acute effects on memory. Taken together these findings may suggest that, in general, repeated consumption of (poly)phenols is required to observe improvements to memory, but that there are specific stages of development which are sensitive to acute manipulation.

A number of mechanisms have been proposed for the action of phenolic compounds on brain function; however, acute cognitive effects of (poly)phenols are commonly attributed to increased cerebral blood flow (CBF). Declines in CBF are observed in ageing [38, 39] and greater cerebral blood flow velocity has been related to significantly less cognitive decline [40], highlighting the importance of CBF modulation to cognition. Increases in CBF parameters have previously been demonstrated following supplementation with flavonoid-rich foods [41, 42], including increased activation in the right middle frontal and right superior parietal cortex following CGJ as measured with fMRI in mildly cognitively impaired adults [22]. The involvement of the fronto-parietal cortical networks in visual attention [43, 44] and the specific role for the right middle frontal and right superior parietal cortex in sustained attention [45, 46] suggest that this increase in activation may relate to the finding of increased speed of attention responses in the current study. However, the lack of attention measures employed by Krikorian et al. [22] and the wide-reaching involvement of the fronto-parietal cortex in other processes not affected here make it difficult to draw firm conclusions at this stage. Importantly, a study of cocoa flavan-3-ol supplementation in older adults, revealed significant benefits to cognition and cerebral blood volume (CBV) in the high flavan-3-ol group relative to control and greater CBV was correlated with better cognitive task performance [47]. Increases to CBF have also been observed in healthy young adults following (poly)phenols [48, 49], demonstrating that these modulations are not restricted to older populations who may be suffering declines in CBF.

One other explanation for the observed improvement to attention reaction time is a modulation of glucose metabolism. Berries have been shown to modulate the glucose and insulin response when compared to matched sucrose and available carbohydrate levels served in water [50, 51]; and to improve the glycaemic profile of bread [52]. Grape seed extract co-consumed with a high-carbohydrate meal has also been shown to lower the post-prandial blood glucose response when measured at 15 and 30-min post-consumption, as well as reducing the area under the curve for the 2-h period of measurement [53]. The impact of glucoregulation on cognition may depend upon the mechanism underlying this effect of (poly)phenols, a number of which have been suggested [16, 54]. However, studies have demonstrated that impaired glucose tolerance is associated with poorer cognition (see [55] for review) and modulation of insulin resistance has been shown to be a significant predictor of cognition following 12-weeks supplementation of cocoa flavan-3-ols, accounting for 17 and 40% change in a composite cognition score in healthy and MCI elderly, respectively. In light of this, further work is required examining the impact of acute glucoregulatory effects of (poly)phenols in relation to cognitive performance. Given the multifunctional nature of (poly)phenol effects it is likely that all of these mechanisms have a role to play and are also inter-related, with endothelial nitric oxide (NO) representing a key molecule in this relationship [56].

In addition to an improvement to attention reaction time, participants also rated themselves to be significantly calmer following purple grape when compared to placebo. Similar acute improvements have been shown in healthy young adults following epigallocatechin gallate (EGCG), evinced as increased calm and decreased stress ratings [57]. Studies showing alerting effects following cocoa [25], blackcurrant [26], and orange juice [27] provide further evidence for acute effects of (poly)phenols on subjective state. A number of mechanisms potentially underlie the demonstrated effect on calm ratings, including modulation of cortisol levels [58] and interaction with GABA_A_ receptors [59]. Mood effects may also be mediated by suppression of monoamine oxidase-B (MAO-B) activity, which was recently reported to be inhibited by 96% following acute administration of blackcurrant juice in healthy young adults [26]. Although this inhibition was not matched by modulation of calm ratings, anxiolytic effects have been reported following chronic administration of berries to rodents [60] and modulation of MAO activity has been proposed as a mechanism for antidepressant-like effects observed following various (poly)phenols in rats [61–63]. Interestingly, speed on a sustained attention task was also increased by the blackcurrant juice in the study by Watson et al. [26], suggesting a potential role for MAO inhibition in the effects on attention reaction time in the current study. Given the possibility for a common mechanism of action and the potential for cognitive and mood effects to impact on one another, correlations were conducted to assess any relationship between the two. No significant associations were observed, but this may reflect a lack of power to detect such effects.

A particularly interesting outcome from the current study is the impact of drink order on cognitive and mood outcomes both pre- and post-dose. A treatment × order interaction on speed of memory indicated that those who had received purple grape at visit 1 were significantly faster at baseline on visit 2 than on visit 1. Similarly, those who received purple grape first had higher post-dose calm ratings than those who received placebo first. These findings both suggest an enduring positive effect of purple grape that had carried over into the second visit. This suggestion is supportive of previous findings showing carryover effects on verbal recall, executive function and driving ability following a 12-week intervention with CJG [23], as well as higher alertness and concentration in those who consumed CGJ first. The demonstration of carryover effects following a single ingestion suggests that these are the result of learning effects rather than enduring changes to physiological mediators such as CBV or glucoregulation as a result of insufficient washout. Order effects are often not considered in studies of this type and should be included in future studies to verify their importance.

Previous studies of cognitive effects of purple grape in humans have employed CGJ supplied for the purpose of the research and consumed at levels far in excess of that which would conventionally be consumed (up to 621 ml/day), making it difficult to convey a clear message to consumers regarding the required dose to achieve the desired effects. The current study demonstrates that a single serving of a commercially available purple grape juice is sufficient to achieve improvements to both cognition and mood and, together with previous findings of improvements to driving ability, help in communicating the health benefits of purple grape to consumers in a meaningful way. A potential problem with using a product ‘off-the-shelf’ relates to a lack of standardisation, but analysis of the purple grape juice employed, conducted in November 2013, demonstrated strikingly similar levels of anthocyanins and total phenolics to those previously published with the same product [9]. This analysis also served to validate the use of white grape juice as a ‘placebo’ (chosen on the basis of its similarity to purple grape juice in terms of mouth feel, taste and sugar content) as phenolic levels were shown to be very low and the addition of blackcurrant cordial was also shown to have minimal impact on these levels. Limitations of the current study relate to the absence of assessment of habitual (poly)phenol intake and the lack of standardisation of diet prior to testing. In keeping with other facets of the study, this lack of standardisation of diet served to provide an ecologically valid demonstration of effects of purple grape juice akin to that which would be expected when consumed in everyday living. Examination of participant’s food diaries revealed good compliance with instructions to consume the same food and drink on study days and to abstain from food for 1 h, and caffeine for 2 h, prior to testing. The food consumed was generally of a low-(poly)phenol content, with 70% of participants reporting no fruit or vegetable intake and the maximum reported being 1 portion of fruit and 1 portion of vegetable. However, it is important that food intake is standardised prior to intervention in order to minimise interference caused by differing nutrient consumption between participants. It is also particularly important to record habitual (poly)phenol intake as this has the potential to impact on gut microbiota, which in turn impacts on absorption of (poly)phenols [64, 65]. For this reason, the stipulation for a beige diet in studies of health effects of (poly)phenols may underestimate any such effects due to reduced bio-efficacy induced by the diet. Similarly, if glucoregulation is found to be an important mediator in the effects of (poly)phenols on cognition, then this may explain null findings when an overnight fast is implemented.

In conclusion, our study demonstrates an acute enhancement of speed of attention and increased calm ratings following purple grape juice in healthy young adults, adding to a growing body of evidence for cognitive benefits of (poly)phenols. These findings require further examination as attention measures are often overlooked in favour of tasks assessing memory, and mood ratings are often difficult to replicate due to their subjective nature. The mechanisms underlying these effects also require further investigation and future studies should strive to include measures of cerebral blood flow, glucoregulation and MAO activity in conjunction with cognitive and mood measures in order to further elucidate their role.
